# Large language models require a new form of oversight: capability-based monitoring

**DOI:** 10.1038/s41746-026-02740-0

**Published:** 2026-05-15

**Authors:** Katherine C. Kellogg, Bingyang Ye, Yifan Hu, Guergana K. Savova, Byron Wallace, Danielle S. Bitterman

**Affiliations:** 1 MIT Sloan School of Management, Boston, MA USA; 2 AI in Medicine Program, Mass General Brigham, Harvard Medical School, Boston, MA USA; 3 Department of Radiation Oncology, Brigham and Women’s Hospital/Dana-Farber Cancer Institute, Boston, MA USA; 4 Department of Computer Science, Brandeis University, Waltham, MA USA; 5 Harvard John A. Paulson School Of Engineering And Applied Sciences, Cambridge, MA USA; 6 Computation Health Informatics Program, Boston Children’s Hospital, Harvard Medical School, Boston, MA USA; 7 Khoury College of Computer Sciences, Northeastern University, Boston, MA USA

**Keywords:** Computational biology and bioinformatics, Engineering, Health care, Mathematics and computing

## Abstract

Large language models (LLMs) have been rapidly adopted in healthcare, but oversight strategies are lacking. We propose capability-based monitoring, motivated by the fact that LLMs are generalist systems whose overlapping internal capabilities are reused across numerous downstream tasks. This approach organizes monitoring around shared capabilities to enable cross-task detection of systemic weaknesses, long-tail errors, and emergent behaviors. We describe considerations for developers, organizational leaders, and professional societies. and policymakers.

## Introduction

The enthusiasm and rapid uptake of generalist artificial intelligence (AI) models, in particular, large language models (LLMs), in healthcare have spurred much discussion of their evaluation and oversight for clinical applications. However, less attention has been paid to the core assumptions about model performance degradation that underpin monitoring strategies, but that break down in the case of LLM use.

To address this, we propose a new capability-based monitoring framework that is better aligned with how LLMs are trained and used in practice. Capability-based monitoring is an organizing principle for generalist LLM oversight that tracks shared model functions across downstream tasks such as summarization or information retrieval, enabling cross-task detection of systemic weaknesses, long-tail errors, and emergent behaviors. We detail (1) a preliminary taxonomy of LLM capabilities, (2) monitoring dimensions and potential metrics, (3) implementation and governance implications of adopting this framework, and (4) future research directions to fully realize the potential of capability-based monitoring. We provide technical recommendations for methodologists and researchers alongside organizational considerations for healthcare leaders and policymakers.

## Why task-based monitoring fails for LLMs

**Fig. 1:**
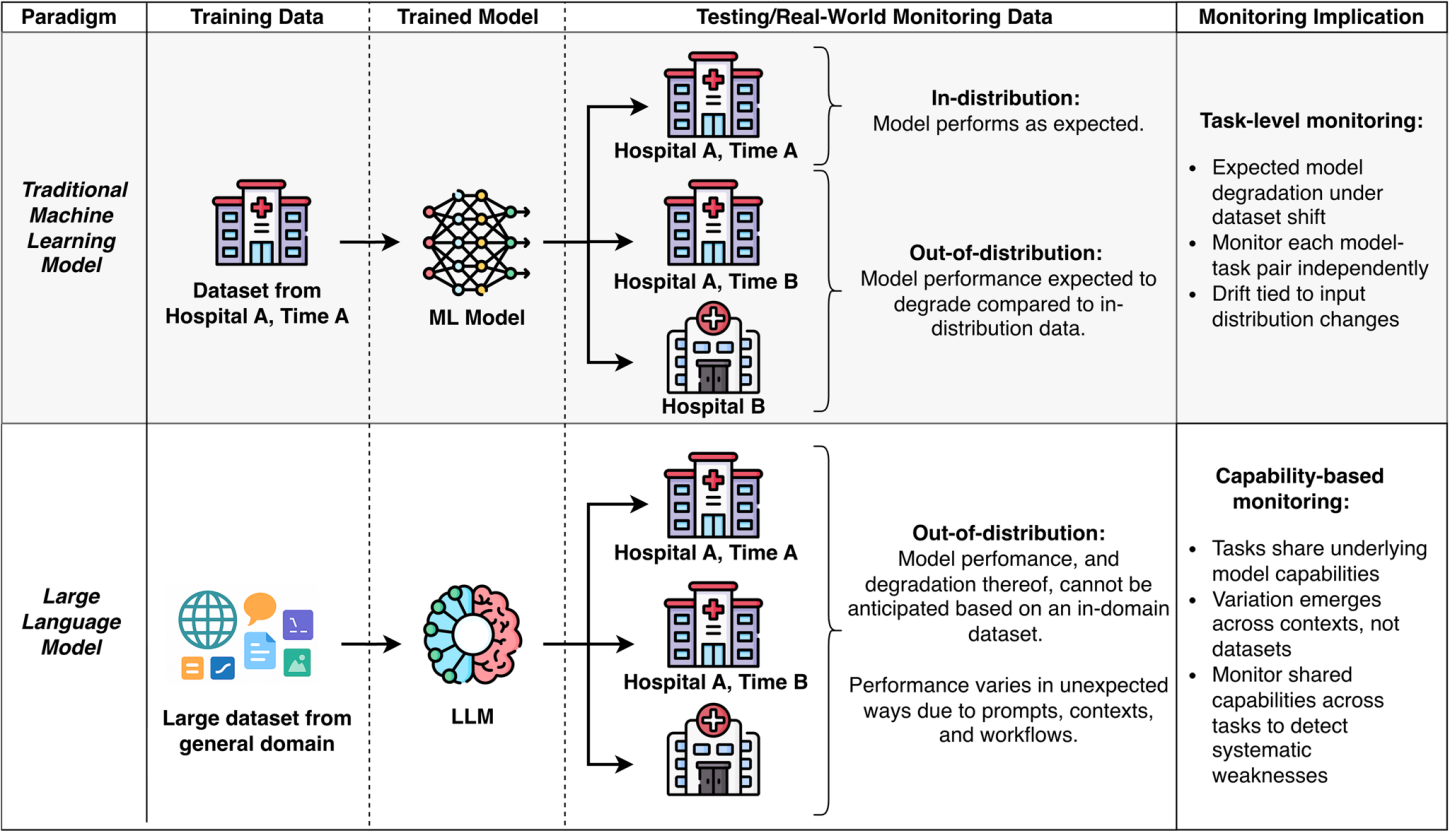
Illustration of train and test data distributions in traditional Machine Learning (ML) models vs. Large Language Models (LLMs). In traditional ML models, it is assumed that test data come from the same underlying distribution (i.e., in distribution; Hospital A, Time A in the figure). As models are applied to different real-world data distributions such as evolution over time (e.g., Hospital A, Time B) and/or new settings (e.g., Hospital B), performance optimized and reported on in-distribution data is no longer reliable. Instead, performance is anticipated to degrade due to overfitting. In LLMs, models are trained from large, general datasets and learn general abilities. All clinical datasets are out of distribution, and traditional notions of ML overfitting and population drift do not straightforwardly apply.

Traditionally, AI implementations in healthcare have been focused on bespoke Machine Learning (ML) models, each trained for a single task using datasets from defined, bounded populations (Fig. 1, ML Paradigm). These models assume that training and test data come from the same underlying distributions. When this assumption is violated, overfitting occurs, leading to degraded performance on new datasets^1,2^. ML models trained for a particular task, such as sepsis prediction^3^, on bounded, labeled clinical datasets reflecting (hopefully) their target clinical population, will always be overfit (that is, only performant for the task and populations they were trained on) to some extent^3,4^. Because of this, performance degradation of ML models post-deployment is a given: models will always degrade because populations and outcome distributions inevitably change compared to the data the model was trained on^1,2^. This has led, sensibly, to model-specific post-deployment monitoring for expected degradation.

In contrast, the emergence of generalist LLMs fundamentally challenges these prior assumptions, driving performance monitoring. Despite not being trained using in-distribution clinical data or specifically for clinically-relevant tasks, LLMs can still capably summarize clinic visit transcripts into note drafts (ambient documentation)^5,6^, answer clinical questions^7^, translate patient instructions^8^, and more. Inevitable model degradation due to changes in populations compared to the training dataset cannot be assumed, because LLMs were not trained for any specific task in any given population (Fig. 1, LLM Paradigm). Many—probably most—clinical tasks will be “out-of-distribution” for the LLM training set, which is massive and often unknown anyway. Thus, traditional notions of ML overfitting and performance degradation do not straightforwardly apply to LLMs. Performance variation due to LLM “overfitting” now occurs due to changes in contextual factors such as the knowledge, culture, deployment environments, or prompting structures, rather than changes from the training dataset distributions defined by input features and labels. While we shouldn’t anticipate degradation due to overfitting in the traditional sense, we do expect that an LLM will behave differently across populations in ways that are not necessarily predictable.

The generalist properties of LLMs make them powerful and drive uptake, but also complicate monitoring. Accordingly, for LLMs and other similar generalist models, monitoring frameworks must evolve. Ongoing task-based oversight is not only impractical as LLMs drive task expansion, but also undesirable because it will leave us blind to shared vulnerabilities. We therefore propose a new organizing principle guiding generalist LLM monitoring that is grounded in how these models are developed and used in practice: capability-based monitoring.

## What is capability-based monitoring?

**Fig. 2:**
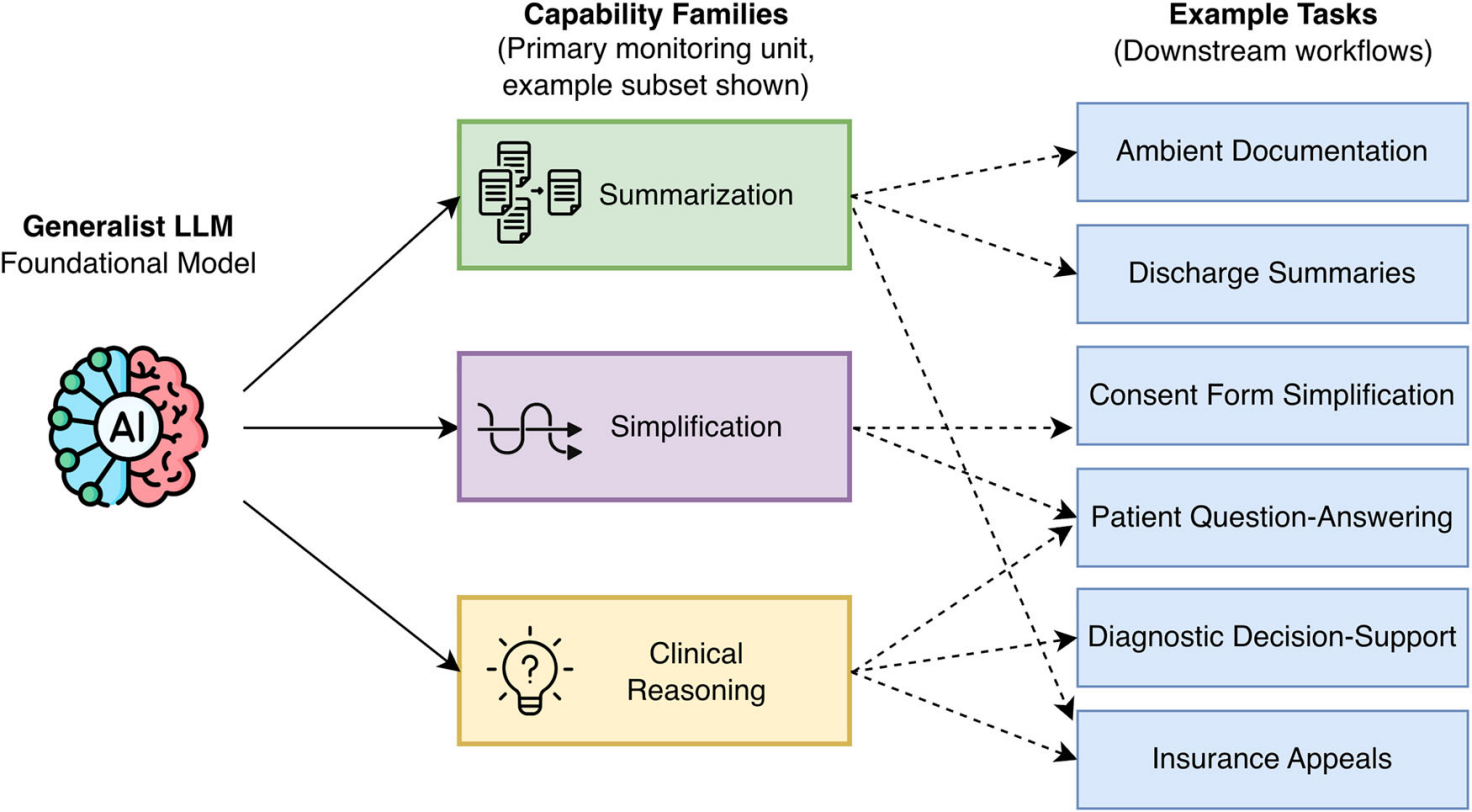
Conceptual monitoring stack for capability-based oversight of clinical large language model (LLM) systems. This conceptual stack organizes monitoring around three layers: a generalist LLM foundation model, intermediate capability families (the primary monitoring unit), and downstream clinical tasks/workflows. Because multiple tasks draw on shared capabilities, monitoring signals can be aggregated at the capability layer across tasks rather than tracked separately for each task. Only an example subset of capability families and tasks is illustrated to demonstrate shared capabilities and instances of tasks using more than one capability.

Capability-based monitoring–monitoring models around shared model capabilities, such as summarization, reasoning, or translation, in order to enable cross-task detection of systemic weaknesses, long-tail errors, and emergent behaviors–is motivated by the fact that LLMs are generalist systems whose overlapping internal capabilities are reused across numerous downstream tasks (Table 1, Fig. 2). We define capabilities as general-purpose LLM functions such as summarization that are the core measurable building blocks for many different tasks. In this approach, capabilities are the unit of monitoring: Tasks relying on the same underlying model and drawing on similar capabilities are monitored collectively—acknowledging that some tasks engage multiple capabilities simultaneously. For instance, the ability to summarize underlies a range of tasks with distinct contexts, such as inpatient discharge summary generation, outpatient pre-charting, and ambient documentation. Monitoring each task in isolation fragments oversight and risks missing cross-cutting vulnerabilities that propagate across tasks. In contrast, capability-based monitoring (Table 2) provides a more practical and comprehensive framework, enabling cross-task evaluation of shared operations, early detection of systemic weaknesses, and identification of edge cases or rare errors that task-specific monitoring might overlook (Fig. 2). This is particularly critical for LLMs, which often struggle with infrequent but clinically significant long-tail scenarios^7^.

**Table 1:** Generalist large language model capability taxonomy

Capability Family	Capability	Core function	Example tasks that require capability
**Content Transformation:** Capabilities focused on generating, modifying, or structuring raw data inputs	Summarization	Ability to compress text, preserve relevant facts, prioritize appropriate facts, and documents	Hospital course summary generation for discharge notes, ambient documentation, discharge instructions, insurance appeals
	Information Extraction	Identify and capture structured data from unstructured documentation Extract discrete fields, classification	Trial matching, medical coding, chart curation, medical coding
	Classification	Assign predefined codes or categories to standardized concepts	Patient portal triage, outcome prediction, medical coding
	Simplification	Generate patient-friendly language (lay and multi-lingual), reply to patients	Patient education, discharge instructions, patient question-answering, consent form simplification
	Translation	Multi-lingual translation and communication	Translation of patient-facing information, real-time translation in clinic
**Clinical knowledge:** Capabilities that use information to inform a user’s decision or answer a query	Clinical Reasoning	Given inputs, provide an appropriate clinical rationale or justification	Clinical decision support, insurance appeals, patient question-answering
	Information Retrieval	Identify and retrieve appropriate documents for an incoming query	Literature search, chart curation
**Governance/Meta-Control:** Capabilities that monitor system self-regulation, safety, and adherence to ethical or operational boundaries.	Factual and Clinical Alignment	Assess adherence to professional and institutional standards and policies; detection of fabricated, hallucinated, or outdated content	Auditing and fact-checking models, often described as LLM-as-judge. Note that these are meta-capabilities, and systems may be designed with embedded controls or separate guardrail models.
	Safety and Refusal Guardrails	Decide when to answer or block a response, safe completion, or refusal of unsafe/uncertain requests	
	Appropriate Language and Tone Monitoring	Detect and filter toxic, stigmatizing, or otherwise inappropriate language	

*LLM* Large Language Model

**Table 2:** Monitoring dimensions and proposed metrics

Monitoring dimension	Description	Example metrics that require human labels	Example automated metrics	Priority monitoring dimensions for each capability							
				Summarization	Information Extraction	Classification	Clinical Reasoning	Simplification	Translation	Information retrieval	Governance/ Meta-Control
Information quality and accuracy	Faithfulness to inputs, factual correctness	% factual errors in sampled outputs scored by a human expert	LLM-as-judge Medical knowledge benchmarks	X	X	X	X	X	X	X	X
Reasoning	Internal logic of output and clinical soundness	Human expert review	LLM-as-judge Medical reasoning benchmarks				X			X	
Style	Clarity, readability, and tone appropriateness	Human expert review	Automated readability metrics LLM-as-judge	X			X	X	X		
Sycophancy* and refusal behavior	Ability to resist declining unsafe or uncertain requests	Human expert review	LLM-as-judge Sycophancy benchmarks				X			X	X
Input robustness and feature drift	Stability under changing prompts, data quality	Human expert review	Overlap metrics LLM-as-judge Text-based statistics (e.g., input tokens)	X	X	X	X	X	X	X	X
Equity	Performance across subgroups (demographics, specialties)	N/A	Distribution of other metrics across subgroups, including raw distribution and fairness metrics	X	X	X	X	X	X	X	X
End-user preference	Human edit rates, acceptance ratios, and escalation frequency	N/A	Edit distance, acceptance rate	X	X	X	X	X	X	X	
Toxicity	Presence of toxic, stigmatizing, or otherwise inappropriate language	Human expert review	LLM-as-judge Toxicity and bias benchmarks	X			X	X	X	X	
Process	Costs, energy, time	N/A	Tokens, costs, FLOPs, latency per unit time (to understand usage), and per query (to understand potential LLM behavior changes)	X	X	X	X	X	X	X	X

*LLM* Large Language Model, *FLOPS* Floating point operations per second

*Sycophancy is defined as excessive agreeableness, at the expense of accuracy and/or safety.

## The proposed framework: capabilities, dimensions, and metrics

LLM behavior depends on intrinsic and extrinsic contextual factors, and LLM performance degradation arises when a model “overfits” these contextual factors that shape its behavior. Intrinsic factors pertain to properties of the model itself, including its alignment with professional standards and values, temporal currency (i.e., how up-to-date its knowledge base is), reasoning quality, robustness to variation in input style or language, and computational efficiency. Extrinsic factors involve human interaction, including the degree of human oversight and the type and extent of human–model collaboration, both of which impact overall system performance^9^. When a model “overfits” these factors, it starts relying too heavily on specific contexts it has seen before, rather than maintaining adaptability. For example, real-world facts and language change over time. If the model is tuned too tightly to outdated knowledge, it struggles with new information. Table 1 outlines a preliminary taxonomy of LLM capabilities, while Table 2 outlines monitoring dimensions (such as reasoning quality and input robustness) and proposed metrics for measuring these intrinsic and extrinsic factors.

Not all dimensions currently validated have automatable monitoring approaches that are known to correlate with human evaluation; many still require human review and gold-standard comparators, although LLM evaluation strategies and metrics, including gaps specific to healthcare, have been extensively discussed in prior work^10–12^. Our framework aims to organize and prioritize metric development and validation. Existing benchmarks in both general and clinical domains, while imperfect, can also supplement real-world monitoring by identifying performance gaps within specific capabilities^13^.

Given the limited availability of validated metrics and ground truth labels they require, the LLM-as-judge paradigm (where a separate model is used to evaluate outputs) is gaining traction as a flexible, extensible monitoring method. We include LLM-as-judge as an automatable metric across several dimensions, but emphasize that these secondary models also require validation and ongoing oversight for each dimension in which they are applied (see Governance/Meta-Control Capability Family, Table 1).

A monitoring strategy should not only identify errors, but also lead to actionable corrections^14^. Importantly, performance degradation across dimensions may not always necessitate a full model update. Limitations arising from intrinsic factors may often be addressed through prompt refinement, improved tool integration, or adjustments to retrieval databases before modifying the underlying model. In contrast, extrinsic factors may call for interventions such as enhanced interface design, user training, or targeted education. We envision primary capability monitoring occurring on a per-LLM basis, as each model is trained on distinct datasets that are typically opaque to the institutions deploying them. Nevertheless, given the shared pretraining corpora and similar tuning paradigms among many LLMs, and the fact that it may not always be known when a vendor updates LLM’s weights, vulnerabilities identified in one model should prompt systematic evaluation across related models.

**Fig. 3:**
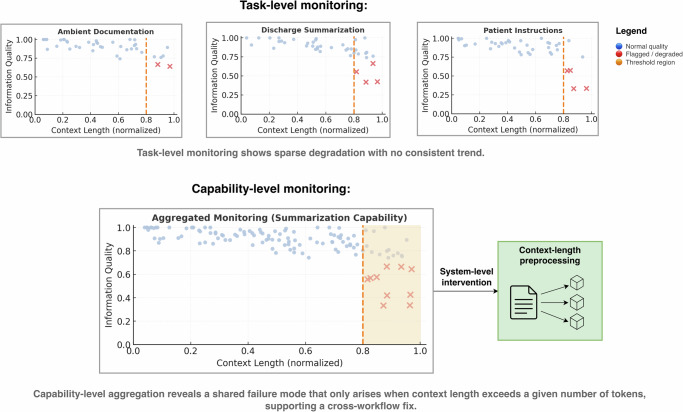
Aggregating task-level signals via capability-based monitoring reveals shared failure modes. Individual monitoring shows sparse quality issues across hospital course discharge summarization, ambient documentation, and patient instructions. Aggregated capability-level monitoring exposes a shared vulnerability, for example, errors arising when context length exceeds a threshold, enabling corrective preprocessing that restores performance across all summarization workflows.

As an example of the strengths of capability-based monitoring, envision an institution that has implemented 3 tasks requiring strong summarization capabilities: hospital course summarization, ambient documentation, and patient-facing discharge instructions (Fig. 3). Missing information is flagged sparsely in all 3 tasks, and the signal only becomes significant when grouped, enabling identification that errors occur when input exceeds a context length threshold, thus a solution is implementation of a new preprocessing step to reduce context length. Similarly, a rare token (wordpiece) repeated many times in a single patient’s inpatient notes is found to trigger biased language. Efficient simulations confirm this is a shared failure mode, so an input filter is implemented for all summarization tasks, avoiding future potential errors for all summarization workflows.

## Implications for developers and methodologists

Implementing capability-based monitoring creates new challenges, implications, and benefits for the healthcare community (Table 3). Key challenges for developers in healthcare organizations include: (a) capability and monitoring dimensions are not yet fully scoped and taxonomized, and will increase over time, (b) it is not feasible to manually monitor all of these metrics for all models, and (c) human oversight and task-specific monitoring will likely still be required for very high-risk applications. Developers can address these challenges by (a) developing visualization-based dashboards and defining evaluation frequency and thresholds for error detection, (b) engaging in two-tiered monitoring with automated screening by LLMs-as-judge and existing automated metrics (high-frequency, low-cost) and human review of flagged cases (low-frequency, high-interpretability), and (c) experimenting with various techniques for addressing human automation bias, over-reliance, and de-skilling in order to provide effective human oversight of models deployed in high-risk tasks.

**Table 3:** Monitoring implementation and oversight design

New Monitoring Challenges with LLMs	Implications for Practice	Benefits	Limitations	Specific Recommendations
**Implications for Developers**				
Task-based monitoring fragments oversight and misses cross-cutting vulnerabilities	• Create registries/ dashboards that visualize performance metrics per capability, not per task	• Scalability and reduced redundancy in compliance and auditing	• Capability and monitoring dimensions not yet fully scoped and taxonomized, and will increase over time	• Develop visualizations of capability-based dashboards • Define evaluation frequency and thresholds for error detection • Audit and log LLM use in a standardized fashion, e.g., using MedLog^15^, extended to include capability family/families for the task
Not feasible to manually monitor all of these metrics for all models	• Implement existing automatic metrics and identify gaps therein • Develop new automated metrics based on identified gaps • New automated metrics may include Judge LLMs: generative models used to evaluate outputs of other LLMs	• Scalable, continuous, and low-cost oversight	• Need to “audit the auditor” via periodic human calibration	• Recommend tiered framework: • Automated screening by Judge LLMs and existing automated metrics (high-frequency, low-cost) • Human review of flagged cases (low-frequency, high-interpretability) • For smaller, more resource-constrained organizations, a “lite” tier with core safety guardrails and summarization monitoring using simple sampling plus manual review may be sufficient
Some truly high-risk implementations will merit their own individualized oversight (e.g., models making treatment recommendations without a human-in-the-loop)	• Maintain risk-stratified evaluation of an emerging technology. High-risk devices still need the appropriate clinical testing before being integrated and monitored • For models that are integrated, there will be a risk threshold at which organizations decide they still need individualized monitoring, but that will be the minority of cases	• Human oversight of very high-risk models	• Humans may miss errors due to automation bias, over-reliance, and de-skilling	• Work with clinicians to investigate the feasibility of llm-as-judge or other monitoring method for a tiered approach over time
Difficult to support the needs of diverse stakeholders (health system leaders, clinical experts, and technical personnel who are distributed across the organization) with a standardized set of metrics	• Identify key stakeholder groups; conduct participatory design sessions with diverse stakeholders to develop prototypes of monitoring dashboards	• Supports teams of health system leaders, clinical experts, and technical personnel that are distributed across the organization as they monitor and respond to model deterioration	• Varied levels of technical expertise and knowledge may limit communication and understanding of metrics • While streamlined compared to task-based monitoring, rapidly expanding capabilities may require ongoing reassessments	• Identify specific data views and functionality required by different stakeholders • Periodically re-evaluate monitoring needs with stakeholders as models advance
Identified performance degradation will need to be addressed	• Develop a standardized approach for root cause analysis • Develop methods for correcting LLM performance • Create back-up strategies for critical LLM-mediated functions	• Enables a resilient model ecosystem that is robust to failures • LLM performance degradation will not always require model fine-tuning; rapid prompt engineering and agentic updates may solve the problem. • Capability-based monitoring enables shared solutions across workflows	• Limited insight and control over vendor LLMs • Increasingly complex agentic systems with tool use and retrieval complicate root cause identification and resolution • The same fix may not always work for all tasks, increasing workload	• Create best practices for manual review of errors, prompt review, and agentic system review • Maintain ongoing communication and collaboration with vendors • Ensure failure is due to LLM itself and not the surrounding architecture, which may be less generalizable • Establish and maintain a database of example inputs for all workflows to confirm shared failure mode and resolution • Maintain shared database of errors and solutions
**Implications for organizational leaders**				
Capability-based monitoring at the business unit-level provides control for business-unit leaders, but risks missing cross-cutting vulnerabilities	• Centralize capability-based monitoring	• Centralized monitoring by capability reduces monitoring burden across hundreds of use cases, and enables cross-context evaluation of shared operations, early detection of systemic weaknesses, and identification of edge cases or rare errors	• Centralization reduces customization of solutions for each business unit and reduces the overall responsiveness to business unit needs • Capability-based monitoring appropriate for post-deployment monitoring is not a substitute for initial needs assessment and evaluation	• Build a team and resources to centralize capability-based monitoring • Identify specific data views and dashboard functionality required by business unit stakeholders. Continue to perform the initial needs assessment and evaluation by model
Merely detecting performance degradation is not sufficient	• Identify who is accountable for diagnosing the root cause of degradation and applying strategies to restore model performance • Develop a set of methods for root cause diagnosis and for restoring model performance • Identify who needs to be informed of model issues, including taking models offline	• Ensures that degradations in model performance will be addressed, and the estimated ROI will continue to be realized	• Limited insight and control over vendor LLMs • Increasingly complex agentic systems with tool use and retrieval complicate root cause identification and resolution • The same fix may not always work for all tasks, increasing the workload	• Maintain ongoing communication and collaboration with vendors • Review failures with business unit leaders to ensure a comprehensive understanding of failures and fixes • Establish collaborations with other institutions to share identified errors and resolutions
Use of LLMs can deskill healthcare workers, making it difficult to take LLMs offline when deterioration is detected	• Institute requirements that professionals regularly practice high-impact tasks without AI, to maintain proficiency • Leverage simulation technology to maintain task proficiency	• Can enable early detection of AI-induced deskilling in high-expertise domains	• Tradeoffs between deskilling solutions that minimize deskilling and those that impose additional time and effort demands	• Institute requirements that professionals regularly practice mission-critical tasks without AI, to maintain proficiency
Lack of clear regulations makes it difficult to determine monitoring metrics	• Integrate with regulatory and accreditation processes by partnering with government affairs teams to create awareness of government agencies/regulatory bodies, and iteratively developing governance policies	• Supports the iterative development of metrics based on changing regulations	• AI technology will continue to move faster than external regulations	• Craft internal governance principles and governance process in advance of regulations • Continue to monitor external regulations to align internal processes with new regulations
Speed of change in models makes it difficult to determine which capabilities should be monitored	• Assign responsibility for external environmental scanning for new model capabilities	• Supports the monitoring of new capabilities • Related capabilities may help anticipate future needs and failure modes • Potential for cross-institution collaboration to learn from others’ experiences	• Automated evaluation metrics will lag behind capability emergence, requiring more intensive initial manual oversight • Emerging capabilities may initially resemble more traditional task-based, single workstream monitoring, which may require bespoke visualizations and metrics • Potential increased computational resource requirements for new models and monitoring thereof create a bottleneck limited by the institutional infrastructure and/or cost	• Establish internal team to review the literature for new capabilities, monitoring methods, and solutions • Institute best practices for integrating a new capability family into the monitoring dashboard • Create a communication structure for developers, informatics, and clinical team members to report gaps in capabilities • Maintain reporting pathways for ad hoc error detection and requirements for critical harm reporting • Create a strategy for prioritizing model assessments to manage computational/cost resources
Individuals may develop LLM implementations for private use and not report these to the organization for monitoring	• Develop pathways and incentives for reporting bespoke workflows to the organization	• Supports monitoring of all models being used by organization members	• Ease of developing new LLM workflows complicates the identification and tracking of all uses • Need for additional resources to identify and integrate uses into centralized monitoring systems	• Reward formalization of models: Provide recognition and rewards for formalizing new models • Increase benefits associated with formalization: Provide resources for integration of models into the EMR system so they can be part of everyday workflow
Comprehensive monitoring introduces privacy and disclosure considerations, particularly for event-level logs	• Create governance standards for the monitoring framework itself, and integrate the system within established security infrastructure	• Centralized and consistent monitoring enables standardized data privacy practices and broad disclosure	• Privacy risks and disclosure needs inherently scale with monitoring, although this is not unique to capability-based monitoring • Event-level logs are useful for auditing, troubleshooting, and accountability, but can contain sensitive information	• Engage legal and ethical bodies, as well as patient stakeholders, to establish institutional standards for disclosure/consent and education around comprehensive monitoring • Include monitoring systems in primary AI governance review • Engage information security teams for unified security review and integration into the data ecosystem
**Implications for professional associations and policymakers**				
Different sophistication in LLM monitoring across institutions	• Develop shared benchmarks and reference frameworks across institutions	• Supports unified registries, clearer accountability, and consistent safety reporting	• In-house technical expertise required • LLM-extrinsic monitoring dimensions may be highly sensitive and unique to specific institutions	• Encourage sharing of frameworks, benchmarking strategies, and other monitoring resources via publication, presentation, and funding opportunities • Formalize working groups and special conferences/workshops for dissemination and training
Inconsistent safety reporting	• Collaborative “monitoring commons” for healthcare AI safety	• Supports unified registries, clearer accountability, and consistent safety reporting	• Institutions must commit to logging LLM use according to shared protocols and taxonomies	• Centralized nationwide database for reporting LLM issues

*LLM* Large Language Model, *ROI* Return on Investment.

## Implications for institutions and policymakers

Key challenges for organizational leaders in healthcare organizations include: a) decentralized capability-based monitoring at the business unit-level gives business unit leaders more control, but risks missing cross-cutting vulnerabilities, b) merely detecting performance degradation through capability-based monitoring is not sufficient, c) individuals may develop LLM implementations via prompt refinement for private use and not report these to the organization for monitoring, and (d) use of LLMs can deskill healthcare workers, making it difficult to take LLMs offline when deterioration is detected. Organizational leaders can address these challenges by (a) centralizing capability-based monitoring while working with business unit stakeholders to identify and create specific data views and functionality required by these decentralized stakeholders, (b) identifying who is accountable for diagnosing the root cause of degradation, and developing a set of methods for root cause diagnosis and restoring model performance, (c) providing recognition, rewards and resources to individuals for formalizing new models, and (d) instituting requirements that professionals regularly practice high-impact tasks without AI, to maintain proficiency.

Addressing these challenges will pay dividends in the future for institutions and regulators. Capability-based monitoring aligns with the EU AI Act’s post-market monitoring requirements for AI-enabled medical devices, which necessitate procedures to track data, assess high-risk system performance, and identify and address emerging risks^15^. Similarly, the US FDA’s lifecycle management approach to AI monitoring will likely incorporate ongoing real-world monitoring, including protocols to identify model and data drift^16,17^.

By measuring consistent metrics and dimensions, capability-based monitoring will support regulators in identifying performance drift and rare error modes at scale. Regulators, payers, and industry should support shared monitoring infrastructure, standardized logging formats, and regional collaboratives, which would be particularly beneficial for resource‑constrained settings.

## Limitations and future research directions

While capability-based monitoring should enable more practical and robust oversight, future work is needed to realize its full potential at scale.

First, our proposed capabilities and metrics are likely not exhaustive, and we encourage the community to contribute to a comprehensive taxonomy of each. As discussed above, there is an ongoing need for development and validation of automated performance metrics relevant to healthcare applications^10–12^. Although more streamlined than task-based monitoring, there are still many ways an organization may wish to visualize capability across models and business units. Research into the optimal visualizations and interface for such monitoring tools will be needed to make sure they are usable and sustainable. Evaluation frequency and thresholds for error detection across all monitoring dimensions will need to be defined and refined as we gain experience implementing LLMs. The quality assurance, process improvement, and statistical quality control fields will play an important role in developing these thresholds.

Second, the monitoring infrastructure itself will have a high cost and resource burden, posing challenges for resource-constrained institutions and institutions without existing informatics infrastructure. Implementing multi-dimensional monitoring across numerous capabilities, particularly when relying on LLM-as-judge models, requires substantial, specialized computational resources and personnel that may be prohibitive. Furthermore, scalability is limited by dependence on human labeling for ground truth to validate and audit metrics, which poses a constraint on the rate at which new dimensions can be validated and implemented. Further, extensive monitoring itself introduces expanded needs for data privacy and disclosure/consent. Regulator, payer, and industry support for shared monitoring would offset burdens.

Because many institutions will use the same underlying LLMs for various tasks, there is an enormous opportunity to extend this strategy to a collaborative monitoring commons across institutions. While capability-based collaborative monitoring will not require sharing data or models, it will require standardized documentation and logging of LLM use. Active uptake and expansion of efforts such as MedLog, a protocol for event-level clinical AI logging, to include capability tagging and associated metadata, will be critical in realizing this vision^18^.

## Conclusions

In the LLM era, “overfitting” in healthcare AI has shifted from model training to prompt, context, and workflow over-adaptation, making the traditional distinction between in-distribution and out-of-distribution clinical data far less predictive of performance. Monitoring of generalist AI, exemplified by LLMs, should be fit-for-purpose: designed to address how LLMs are trained and used in practice, not simply extended from traditional models that have different performance and generalization assumptions. As such, the unit of monitoring must evolve from tasks to capabilities, tracking shared behaviors across contexts. Capability-based monitoring is at once technically necessary and organizationally scalable. Healthcare systems, vendors, and regulators should adopt capability-based frameworks to ensure safe, equitable, and sustainable deployment of generalist AI.

## Data Availability

No datasets were generated or analyzed during the current study.

## References

[1] Wong, A. & Sussman, J. B. Understanding model drift and its impact on health care policy. *JAMA Health Forum* **6**, e252724 (2025). 40815525 10.1001/jamahealthforum.2025.2724

[2] Finlayson, S. G. et al. The Clinician and Dataset Shift in Artificial Intelligence. *N. Engl. J. Med.* **385**, 283–286 (2021). 34260843 10.1056/NEJMc2104626 PMC8665481

[3] Wong, A. et al. External validation of a widely implemented proprietary sepsis prediction model in hospitalized patients. *JAMA Intern. Med.* **181**, 1065–1070 (2021). 34152373 10.1001/jamainternmed.2021.2626 PMC8218233

[4] Habib, A. R., Lin, A. L. & Grant, R. W. The epic sepsis model falls short-the importance of external validation. *JAMA Intern. Med.* **181**, 1040–1041 (2021). 34152360 10.1001/jamainternmed.2021.3333

[5] Afshar, M. et al. A Novel Playbook for Pragmatic Trial Operations to Monitor and Evaluate Ambient Artificial Intelligence in Clinical Practice. *NEJM AI* **2**, 10.1056/aidbp2401267 (2025). 10.1056/aidbp2401267 PMC12435388 40959192

[6] You, J. G. et al. Ambient documentation technology in clinician experience of documentation burden and burnout. *JAMA Netw*. *Open* **8**, e2528056 (2025). 10.1001/jamanetworkopen.2025.28056 PMC12371510 40839265

[7] Chen, S. et al. The effect of using a large language model to respond to patient messages. *Lancet Digit. Health* **6**, e379–e381 (2024). 38664108 10.1016/S2589-7500(24)00060-8 PMC11829255

[8] Ray, M. et al. Evaluating a large language model in translating patient instructions to Spanish using a standardized framework. *JAMA Pediatr.* **179**, 1026–1033 (2025). 40622720 10.1001/jamapediatrics.2025.1729 PMC12235533

[9] Sujan, M. et al. Human factors challenges for the safe use of artificial intelligence in patient care. *BMJ Health Care Inform.* **26**, e100081 (2019). 10.1136/bmjhci-2019-100081 PMC7252977 31780459

[10] Agrawal, M., Chen, I. Y., Gulamali, F. & Joshi, S. The evaluation illusion of large language models in medicine. *NPJ Digit. Med.* **8**, 600 (2025). 41057566 10.1038/s41746-025-01963-x PMC12504413

[11] Moreno, A. C. & Bitterman, D. S. Toward clinical-grade evaluation of large language models. *Int. J. Radiat. Oncol. Biol. Phys.* **118**, 916–920 (2024). 38401979 10.1016/j.ijrobp.2023.11.012 PMC11221761

[12] Sai, A. B., Mohankumar, A. K. & Khapra, M. M. A survey of evaluation metrics used for NLG systems. *ACM Comput. Surv.* **55**, 1–39 (2023).

[13] Ni, S. et al. A survey on large language model benchmarks. *ArXiv* abs/2508.15361, (2025).

[14] Guan, H., Bates, D. & Zhou, L. Keeping Medical AI Healthy and Trustworthy: A Review of Detection and Correction Methods for System Degradation. In *IEEE Transactions on Biomedical Engineering*. 10.1109/TBME.2025.3642706 (2025). 10.1109/TBME.2025.3642706 PMC13050583 41370148

[15] EU Artificial Intelligence Act. https://artificialintelligenceact.eu/.

[16] Request For Public Comment: Measuring and Evaluating Artificial Intelligence-enabled Medical Device Performance in the Real-World. U.S. Food and Drug Administration https://www.fda.gov/medical-devices/digital-health-center-excellence/request-public-comment-measuring-and-evaluating-artificial-intelligence-enabled-medical-device?utm_medium=email&utm_source=govdelivery (2025).

[17] Warraich, H. J., Tazbaz, T. & Califf, R. M. FDA perspective on the regulation of artificial intelligence in health care and biomedicine. *JAMA* **333**, 241–247 (2025). 39405330 10.1001/jama.2024.21451

[18] Noori, A. et al. A global log for medical AI. *arXiv [cs.AI]* (2025).

